# Bayesian probit regression model for the diagnosis of pulmonary fibrosis: proof-of-principle

**DOI:** 10.1186/1755-8794-4-70

**Published:** 2011-10-05

**Authors:** Eric B Meltzer, William T Barry, Thomas A D'Amico, Robert D Davis, Shu S Lin, Mark W Onaitis, Lake D Morrison, Thomas A Sporn, Mark P Steele, Paul W Noble

**Affiliations:** 1 Department of Medicine, Division of Pulmonary, Allergy and Critical Care Medicine, Department of Medicine, Duke University Medical Center, Durham, North Carolina, USA; 2 Department of Biostatistics and Bioinformatics, Duke University Medical Center, Durham, North Carolina, USA; 3 Institute for Genome Science and Policy, Duke University Medical Center, Durham, North Carolina, USA; 4 Department of Surgery, Division of Cardiovascular and Thoracic Surgery, Duke University Medical Center, Durham, North Carolina, USA; 5 Department of Immunology, Duke University Medical Center, Durham, North Carolina, USA; 6 Department of Pathology, Duke University Medical Center, Durham, North Carolina, USA

## Abstract

**Background:**

The accurate diagnosis of idiopathic pulmonary fibrosis (IPF) is a major clinical challenge. We developed a model to diagnose IPF by applying Bayesian probit regression (BPR) modelling to gene expression profiles of whole lung tissue.

**Methods:**

Whole lung tissue was obtained from patients with idiopathic pulmonary fibrosis (IPF) undergoing surgical lung biopsy or lung transplantation. Controls were obtained from normal organ donors. We performed cluster analyses to explore differences in our dataset. No significant difference was found between samples obtained from different lobes of the same patient. A significant difference was found between samples obtained at biopsy versus explant. Following preliminary analysis of the complete dataset, we selected three subsets for the development of diagnostic gene signatures: the first signature was developed from all IPF samples (as compared to controls); the second signature was developed from the subset of IPF samples obtained at biopsy; the third signature was developed from IPF explants. To assess the validity of each signature, we used an independent cohort of IPF and normal samples. Each signature was used to predict phenotype (IPF versus normal) in samples from the validation cohort. We compared the models' predictions to the true phenotype of each validation sample, and then calculated sensitivity, specificity and accuracy.

**Results:**

Surprisingly, we found that all three signatures were reasonably valid predictors of diagnosis, with small differences in test sensitivity, specificity and overall accuracy.

**Conclusions:**

This study represents the first use of BPR on whole lung tissue; previously, BPR was primarily used to develop predictive models for cancer. This also represents the first report of an independently validated IPF gene expression signature. In summary, BPR is a promising tool for the development of gene expression signatures from non-neoplastic lung tissue. In the future, BPR might be used to develop definitive diagnostic gene signatures for IPF, prognostic gene signatures for IPF or gene signatures for other non-neoplastic lung disorders such as bronchiolitis obliterans.

## Background

Pulmonary fibrosis is a significant cause of morbidity and mortality worldwide [1,2]. The multiple subtypes of pulmonary fibrosis carry different prognoses. Idiopathic pulmonary fibrosis (IPF), for example, is a particularly fatal subtype of pulmonary fibrosis that leads to death within 3-5 years of its diagnosis; IPF does not usually respond to immunosuppressant therapy [3-5]. Nonspecific interstitial pneumonia (NSIP) is another subtype of pulmonary fibrosis that has much better rates of survival and treatment response [2,6]. All together, there are perhaps 200 subtypes of pulmonary fibrosis [7]. The American Thoracic Society and European Respiratory Society published a classification scheme that describes the major subtypes of pulmonary fibrosis [2]. Other authors describe complex algorithms for making an accurate diagnosis of pulmonary fibrosis [7-9].

An accurate diagnosis of pulmonary fibrosis requires the integration of clinical, radiographic and pathologic information [3,10]. Yet, there is no single test by which an accurate diagnosis of pulmonary fibrosis can be secured. The complexity of diagnostic algorithms makes it difficult to establish an accurate diagnosis of pulmonary fibrosis outside of the academic setting [11,12]. This increases the risk for inaccurate diagnoses and the administration of inappropriate treatments. For the purposes of this study, we focused on IPF. The goal of this study was to assess methods by which a diagnostic test for IPF could be developed.

Bayesian probit regression (BPR) is a statistical method, well-suited to the analysis of highly dimensional data such as that produced by gene expression profiling. In the past, BPR was used to model differences in gene expression detected in cases of prostate cancer and ovarian cancer [13,14]. BPR has never been used to analyze non-neoplastic lung tissue.

The experiments described herein were designed as a proof-of-principle for the concept of "developing IPF gene expression signatures with BPR". Our aims were to develop a provisional diagnostic model for IPF; and to establish BPR as an appropriate method for developing additional gene signatures for non-neoplastic lung disease.

## Methods

### Ethics Statement

This study was approved by the Duke University Health System Institutional Review Board (IRB # Pro00007903, Pro00008725 and Pro00008819) and written informed consent was obtained from all subjects.

### Study Population

We selected consecutive patients with IPF. Specimens were collected from 11 patients. All cases fulfilled multidisciplinary diagnostic criteria described in the American Thoracic Society/European Respiratory Society consensus statement [3]. In addition, pathological confirmation was obtained for every case. IPF was confirmed by the identification of a usual interstitial pneumonia (UIP) under the light microscope.

Samples of whole lung tissue were obtained at the time of diagnostic surgical lung biopsy (6 cases) or during orthotopic lung transplantation surgery (5 cases). Specimens were collected from both the upper and lower lobes whenever possible (6 out of 11 cases).

Control specimens (6 cases) were obtained from donated organs that were accepted for lung transplantation. At the end of lung transplant surgeries, we collected a portion of the newly transplanted lung that was removed during the process of routine lung volume reduction.

### Sample Processing

Samples were immediately processed following removal from the body. First, specimens were cut into small pieces (< 5 mm in diameter), immersed in RNAlater solution (Ambion, Inc., Austin, TX) and incubated overnight at 4°C as per the manufacturer's instructions. Next, the supernatant was removed and samples were stored in a -20°C freezer.

At a later date, frozen RNA-protected samples were homogenized with a FastPrep device by using Lysing Matrix A (MP Biomedicals, Solon, OH). Total RNA was extracted from the homogenates by using RNAqueous-4PCR kits (Ambion, Inc., Austin, TX) as per the manufacturer's instructions. RNA quantity was measured with a spectrophotometer and RNA quality was assessed with a bioanalyzer (Agilent Technologies, Santa Clara, CA).

Isolated RNA was used to produce labeled-cRNA. Then labeled-cRNA was hybridized to Affymetrix Human Genome U133 Plus 2.0 GeneChips; and scanned using standard Affymetrix protocols. Our complete dataset is available through the Gene Expression Omnibus database (http://www.ncbi.nlm.nih.gov/geo/; accession number GSE24206).

### Validation Cohort

The dataset for the validation cohort was accessioned from the Gene Expression Omnibus (http://www.ncbi.nlm.nih.gov/geo/; accession number GSE10667). This dataset contains raw and processed gene expression profiles from thirty-one patients with IPF and 15 expression profiles from normal lung controls. This data was contributed to the Gene Expression Omnibus by investigators at the University of Pittsburgh; these samples were previously described [15,16]. This dataset was generated on Agilent-014850 Whole Human Genome 4 × 44K Microarrays according to the manufacturer's protocol as reported by the original investigators.

### Statistical Analysis

#### Data processing

Expression estimates for the Affymetrix U133 Plus 2.0 GeneChips were obtained by robust multi-array average (RMA) then log_2 _transformed [17-19]. Data were filtered prior to analysis to annotated probe sets with average expression values > 4.

#### Unsupervised cluster analysis

Global patterns of gene expression were evaluated (with the top 10% of genes by coefficient of variation) by Principal Component Analysis (PCA) and hierarchical clustering algorithms using the average linkage of the Pearson correlation coefficient.

#### Differential gene expression

Paired t-tests were used to assess differences in gene expression between upper and lower lobe samples. Unpaired Student's t-tests were used to compare the gene expression from IPF biopsies and IPF explants.

#### Supervised classification

Multi-gene models for binary phenotypes were derived using singular value decomposition (SVD) and Bayesian probit regression models, as described previously [13,14,20]. In tuning the model parameters, a data-driven empirical approach was taken to select the optimal number of features in each gene signature, using the sum of deviances as a metric of relative performance. For a complete description, refer to Additional file 1, Supplemental Methods and Additional file 2, Figure S1.

#### Validation

To independently validate the multi-gene models, features were mapped on a many-by-many basis between the training dataset (Affymetrix HGU133 Plus 2.0) and GSE10667 dataset (Agilent-014850 Whole Human Genome 4 × 44K Microarray) using Unigene and RefSeq IDs (Additional files 3, 4 and 5, Tables S1-S3). Gene expression estimates were scale/shift normalized across the datasets, and loadings from the SVD were derived from the training dataset only, such that predicted probabilities from the Bayesian regression model are independent for the validation set. Association with the phenotype of IPF versus normal control was assessed using a Wilcoxon rank sum test, and the predictive value of the signature was evaluated using receiver operator characteristic (ROC) curves.

### Computational Software

All microarray pre-processing, BPR modeling and analyses were performed using R version 2.9 and Bioconductor packages designed for use with Affymetrix microarray data (Additional file 6, Software Codes). Graphical images were produced in R and in MATLAB R2009a (The MathWorks, Inc., Natick, MA).

## Results

### Patients

Demographic and physiologic characteristics of the 11 patients enrolled in this study are reported in Table 1. Each patient underwent either a medically-indicated surgical lung biopsy or medically-indicated lung transplantation surgery; remnants of the biopsy sample or pieces of the explanted lung were preserved for microarray analysis. Physiologic measurements were made prior to surgery. When we compared biopsy to explant, we found no differences in the average age of patients (60.67 ± 2.72 to 66.6 ± 0.68); the proportion of males (83% versus 60%); or the forced vital capacity (65.17 ± 5.75 to 56.8 ± 5.54). However, diffusing capacity for carbon monoxide was decreased in patients undergoing lung transplantation surgery (61.83 ± 6.38 to 29.2 ± 4.19, p-value < 0.01) which is statistically significant in this patient cohort.

**Table 1:** Study Population

Patient Number	Sample ID	Age	Gender	FVC%	DLCO%	Sample Type	Multiple Lobes Sampled?
1	Biopsy_140U	58	Male	55	54	Biopsy	No
2	Biopsy_142U	56	Female	55	65	Biopsy	No
3	Biopsy_144U	70	Male	84	87	Biopsy	No
4	Biopsy_145U	54	Male	68	52	Biopsy	No
5	Biopsy_149U	58	Male	79	70	Biopsy	Yes
	Biopsy_149L						
6	Biopsy_159U	68	Male	50	43	Biopsy	Yes
	Biopsy_159L						

7	Explant_146L	64	Male	56	23	Explant	No
8	Explant_152U	67	Male	53	29	Explant	Yes
	Explant_152L						
9	Explant_157U	67	Male	51	34	Explant	Yes
	Explant_157L						
10	Explant_158U	68	Female	78	18	Explant	Yes
	Explant_158L						
11	Explant_160U	67	Female	46	42	Explant	Yes
	Explant_160L						

FVC% = forced vital capacity (expressed as a percentage of the normal expected value); DLCO% = diffusion capacity for carbon monoxide (expressed as a percentage of the normal expected value).

### Global Analysis of Gene Expression

**Figure 1:**
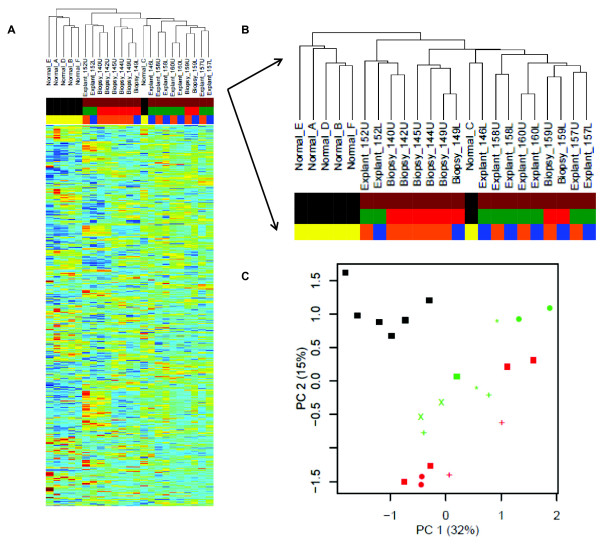
**Unsupervised cluster of the complete dataset (training cohort)**. Samples include normal lung (*black*) and IPF (*brown*). IPF is divided into biopsy (*red*) and explant (*green*). Samples are also identified by their lobe of origin: upper lobe (*orange*), lower lobe (*blue*) or unknown lobe (*yellow*). **(A) **Unsupervised hierarchical clustering of all samples based on gene expression profiles. Samples include 6 normals (Normal_[A through F]) and 17 samples of IPF (6 upper/lower lobe pairs and 5 singletons) of which 8 are biopsies (Biopsy_[3-digit sample ID][U = upper or L = lower] and 9 are explants (Explant_[3-digit sample ID][U = upper or L = lower]). **(B) **Enlargement of the dendrogram, sample names and color key from Figure 1A. **(C) **Samples are plotted according to expression of the first two Principal Components. [Key: singletons = colored square; all other shapes represent lobar pairs].

To explore gene expression differences (and similarities) between all of the samples, we carried out an unsupervised hierarchical cluster of the entire dataset (Figure 1A). The dataset contains gene expression from 23 samples: 17 samples of IPF from 11 different patients (6 pairs of samples from upper and lower lobes; and 5 samples of single lobes); and 6 samples from normal lung donors. Examining the hierarchical dendrogram (Figure 1B), we found a natural separation between IPF samples and normal lung samples (normals are found on the left-hand side of the figure; IPF samples fall in the middle and on the right-hand side of the dendrogram), with the exception of one outlier, a sample of normal lung (Normal_C) which falls among the IPF samples.

We further observed that pairs of samples from the upper and lower lobes have similar global gene expression profiles, such that each pair forms its own node in the hierarchical cluster. In order to meet the assumptions of independent and identically distributed samples for developing signatures of IPF, we chose to use only one sample (the upper lobe, when available) per patient in the subsequent analyses.

Finally, we observed that explanted samples and biopsied samples largely segregate in the hierarchical clusters with the exceptions of: one pair of biopsied samples (Biopsy_159U and Biopsy_159L) and one normal sample (Normal_C) falling in the explant cluster; and a pair of explants (Explant_152U and Explant_152L) which fall in the biopsy cluster.

To further evaluate global differences in gene expression, we decomposed the high-dimensional gene expression data using principal component analysis (PCA), whereby 47% of the variance in this dataset is captured within the first two principal components for all 23 samples. Again, we found that normal and IPF samples are distinctive (Figure 1C). Furthermore, a separation was seen between the biopsied IPF samples and the explants. Meanwhile, the upper/lower lobe pairs showed strong similarity (average Pearson correlation of 0.929) as compared to unmatched pairs (average Pearson correlation of 0.781).

### Comparing Gene Expression from the Upper and Lower Lobes

**Figure 2:**
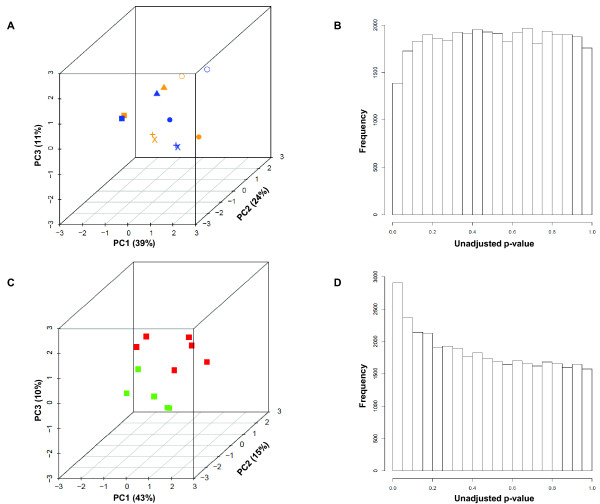
**Comparison of samples from different lobes; comparison of samples from biopsy and explant**. In panels A and B, we compare samples from the upper (*orange*) and lower lobes (*blue*). In panels C and D, we compare samples obtained by biopsy (*red*) versus explant (*green*). **(A) **Upper and lower lobe samples are plotted according to expression of the first three Principal Components. [Key: each shape represents a lobar pair.] **(B) **Paired LIMMA tests were performed for every gene to compare expression between the upper and lower lobes; a frequency histogram shows the distribution of unadjusted p-values. **(C) **Biopsied and explanted samples are plotted according to expression of the first three Principal Components. **(D) **Unpaired LIMMA tests were performed for every gene to compare expression between biopsies and explants; a frequency histogram shows the distribution of unadjusted p-values.

To further characterize the upper/lower lobe pairs, we decomposed the gene expression data for pairs alone by PCA. This analysis captured 74% of the variance within the first three principal components. We plotted the upper/lower lobe pairs according to expression of the first three principal components (Figure 2A) and found that clusters were not determined by lobe, but rather by the patient (intraclass correlation coefficient = 0.474, p-value = 0.02 [for the first principal component]).

To identify genes that might be differentially expressed between the upper and lower lobes, we performed a paired LIMMA test [21,22] as an empirical Bayesian approach to analyzing microarray data that uses hierarchical linear models to improve estimates of variance. First, we excluded unannotated and lowly expressed genes. Then we plotted the unadjusted p-values for all tests on a frequency histogram and note that the frequency of nominally significant p-values (< 0.05) is no greater than that expected by chance alone (Figure 2B). This suggests that greater differences in expression are observed across subjects than between upper and lower lobe, as supported by serial 2-way ANOVA (data not shown), and the hierarchical cluster in Figure 1 where 5 of 6 pairs are noted to be most similar. Therefore, a single sample from each patient was selected for further analysis regardless of lobe.

### Comparing Gene Expression from Biopsies and Explants

To investigate the difference between biopsies and explants, we selected the data from this subset of samples (excluding lobar replicates) and decomposed the data by PCA such that 68% of the variance was captured within the first three principal components. The samples were plotted according to expression of the first three principal components (Figure 2C). Here, we could appreciate a distinct separation between IPF biopsies and IPF explants.

Next, we carried out the LIMMA test to identify genes that were differentially expressed between biopsy and explant. Before adjusting p-values, we plotted the results on a frequency histogram. We noted that the frequency of nominally significant p-values (< 0.05) was greater than expected by chance alone (Figure 2D). After adjusting the p-values with the Benjamini-Hochberg step-down method to control the false discovery rate (FDR) [23], 13 probesets (corresponding to 11 unique genes) were identified as statistically significant using a FDR threshold of 10% (Additional file 7, Table S4).

### Approach to Developing Gene Expression Signatures

**Figure 3:**
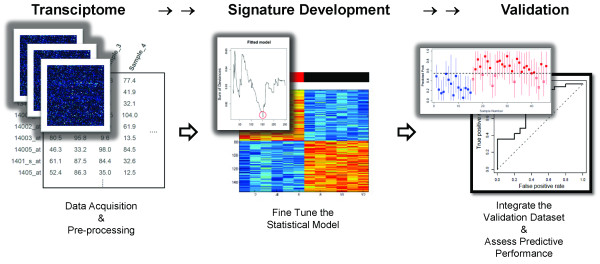
**Schematic diagram of the workflow**.

A schematic diagram illustrates the process by which we develop genomic signatures using BPR models (Figure 3). The first step is to select, as the training dataset, a collection of samples that represent two distinct phenotypes. Prior to analysis, the training dataset is filtered to exclude unannotated and lowly expressed genes, without regard to phenotypic information.

Because there is no prior knowledge on which to base the number of genes included in the model, we propose an iterative data-driven approach to model-fitting. We propose using the "sum of deviances" between observed and predicted phenotypes, coupled with the "misclassification rate" under a leave-one-out process, to determine the optimal size of our BPR model (i.e., the number of genes to include in the regression equation). Once the number of genes is selected, the model is summarized by the gene annotation and the average of the posterior distribution of the linear predictor under the Bayesian model. The gene signature is visualized by a heatmap that shows normalized expression values of the selected genes (rows) over the set of samples (columns).

Finally, a second set of samples is used to test the performance of the tuned model. This represents an independent validation. Because the validation dataset is derived on a different microarray platform, expression values need to be mapped and normalized in a merged dataset to account for differences in batch and the information content of each array. Then, each sample in the validation dataset is applied to the Bayesian regression model in order to generate a predictive probability (from 0.0 to 1.0) as a relative score indicating the likelihood of one phenotype over the other. Given information regarding the true phenotype of each validation sample, it is possible to construct a receiver-operating characteristic (ROC) curve for the predictive value of the gene signature.

### Binary Classification for Signature Development

**Figure 4:**
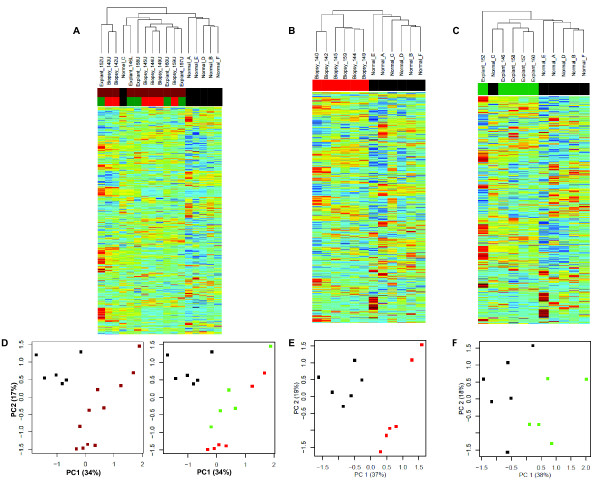
**The training sets**. All IPF (*brown*), IPF biopsy (*red*), IPF explant (*green*) and normal lung (*black*). **(A) **Unsupervised hierarchical clustering of 11 IPF samples (6 biopsies and 5 explants) and 6 normals. **(B) **Unsupervised hierarchical clustering of 6 IPF biopsy samples and 6 normals. **(C) **Unsupervised hierarchical clustering of 5 IPF explants samples and 6 normals. **(D) **11 IPF samples (biopsy and explant) and 6 normals are plotted according to expression of the first two Principal Components. The left panel shows the difference between IPF and normal lung; while the right panel reveals the difference between IPF biopsy and IPF explant. **(E) **6 IPF biopsies and 6 normals are plotted according to expression of the first two Principal Components. **(F) **5 IPF explants and 6 normals are plotted according to their expression of the first two Principal Components.

We chose to develop three separate models for the classification of IPF; we planned to test each model for diagnostic accuracy (i.e., functional validity) in an independent dataset. We developed the first model from all IPF samples (excluding lobar replicates) versus normal controls. This training dataset is summarized in an unsupervised hierarchical cluster (Figure 4A) of the genes showing the largest coefficient of variation (CoV).

Since we identified differential gene expression between IPF biopsies and IPF explants, we chose to separately develop diagnostic signatures from each class, as compared to normal controls. For the IPF biopsy samples, the training dataset is summarized in an unsupervised hierarchical cluster (Figure 4B). Likewise, for the subset of IPF explants, the training dataset is summarized in an unsupervised hierarchical cluster (Figure 4C).

The three training datasets are each decomposed by PCA and the samples are plotted with regard to the first two principal components (Figures 4D,E and 4F).

### Model Parameterization for Signature Development

For all signatures, the top two factors from singular value decomposition were used to fit independent terms to the BPR models. The "misclassification rate" and "sum of deviance" were used to determine the number of genes in each model, as described in Additional file 1 (also see Additional file 2, Figure S1). We determined that 151 genes were needed to optimize the "All IPF" model; 153 genes were needed to optimize the "IPF Biopsy" model; and 70 genes were needed to optimize the "IPF Explant" model.

**Figure 5:**
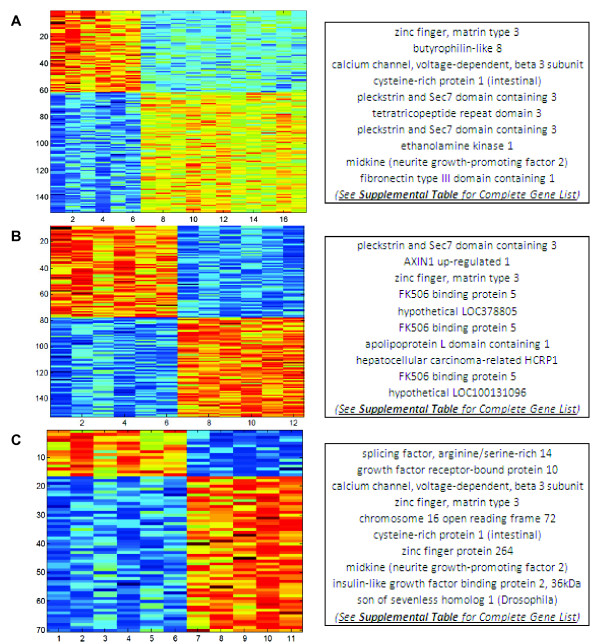
**Gene signatures**. **(A) **A heatmap displays the normalized expression values of 151 genes that comprise the *All IPF *model, derived from 6 normals and 11 IPF samples (rows = genes; columns [left to right] = 6 normals, 6 biopsies and 5 explants). A partial gene list (top ten) is shown to the right. **(B) **A heatmap and partial gene list for the *IPF Biopsy *model, 153 genes derived from 6 normals and 6 IPF biopsies. **(C) **Heatmap and partial gene list for the *IPF Explant *model, 70 genes derived from 6 normals and 5 IPF explants.

BPR was performed on each training dataset. Each model was visualized with a heatmap (Figure 5). To illustrate that each training dataset produces a unique set of predictors, we list the top 10 gene predictors alongside each model. The complete gene list for each signature is supplied in the additional files (see Additional files 8, 9 and 10, Tables S5-S7).

### Independent Validation of Gene Signatures

We used the GSE10667 dataset to test each gene signature. By using the same dataset to validate all three signatures, we were able to make a direct comparison between the models.

First we mapped the features of the Agilent microarray GSE10667 dataset to the corresponding features in our Affymetrix training datasets. We found that 148 features of the GSE10667 dataset mapped to features of the "All IPF" model (out of a possible 151 features, 98.0%); 151 features were mapped to the "IPF Biopsy" model (out of 153 possible features, 98.7%); and 69 features were mapped to the "IPF Explant" model (out of 70 possible features, 98.6%). After features were mapped, we merged the training and validation datasets. Gene expression was normalized across the merged datasets.

**Figure 6:**
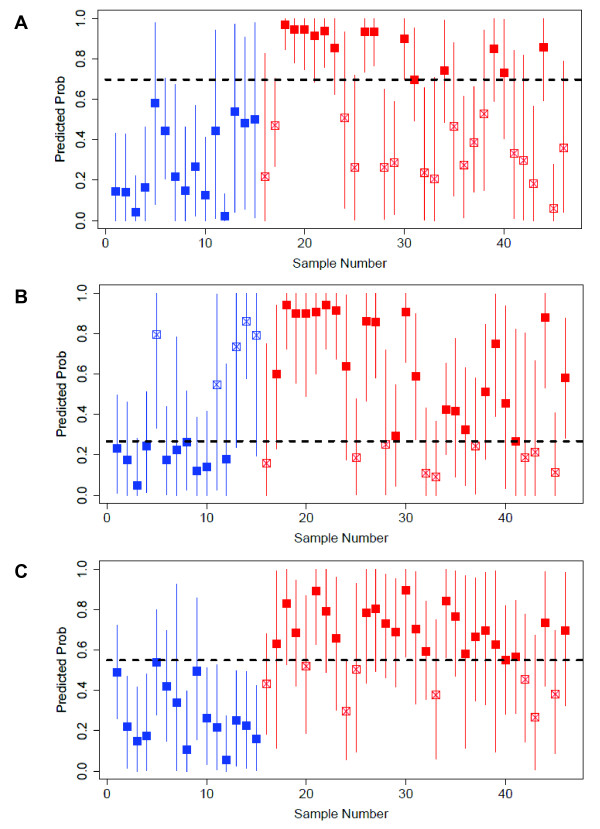
**Validation tests**. Each sample of the GSE10667 cohort is assigned a probability of IPF. Cutoffs were determined by calculating the Youden index. The true phenotype of each sample is indicated in color (15 normals [*blue*] and 31 IPF [*red*]). **(A) **The *All IPF *signature is used to assign IPF probability. **(B) **The *IPF Biopsy *signature is used to assign IPF probability. **(C) **The *IPF Explant *signature is used to assign IPF probability.

Then, each model was used in turn to predict the phenotype of each sample in the validation cohort (Figure 6A, B and 6C). Predicted probabilities indicate the likelihood of IPF. The true phenotype of each validation sample is shown in color (*blue *for normal and *red *for IPF). Correct predictions are indicated with a solid marker while incorrect predictions are indicated with an open marker. The Youden index was used to compute cut points that maximize linear combinations of sensitivity and specificity for each model in this cohort, run on Agilent arrays. Evaluation of the quality of these thresholds would require additional validation on the Agilent platform as part of future investigations.

**Figure 7:**
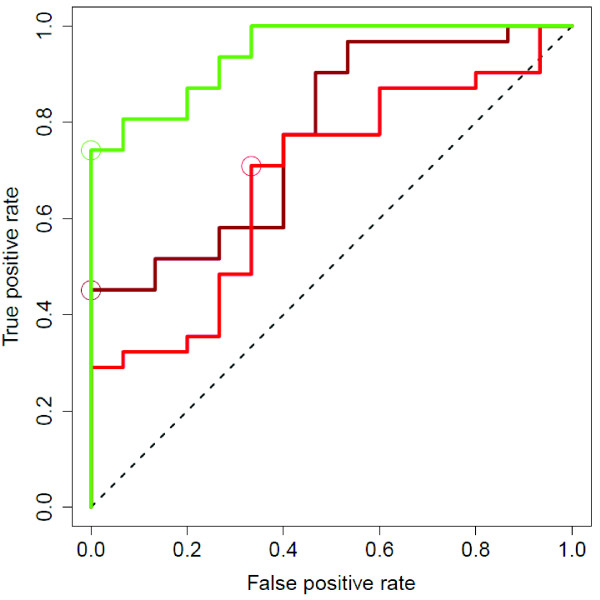
**ROC curves**. All IPF (*brown*), IPF Biopsy (*red*) and IPF Explant (*green*) are shown for comparison. Optimal cutoff points are circled.

ROC curves are drawn on a single graph to facilitate comparison (Figure 7). Area under the curve, sensitivity, specificity, positive and negative predictive values and overall predictive accuracy are reported in Table 2. Wilcoxon rank sum was performed on each signature to test the general association of predictions and phenotypes. Interestingly, the "IPF Explant" model outperforms the "All IPF" and "IPF Biopsy" models.

**Table 2:** Operating Characteristics of the Gene Signatures

Gene Signature Model	Area Under the Curve	Sensitivity	Specificity	Positive Predictive Value	Negative Predictive Value	Overall Accuracy	Wilcoxon Rank-sum (p-value)
All IPF	0.774	45%	100%	100%	47%	63%	0.0023
IPF Biopsy	0.682	71%	67%	81%	53%	70%	0.048
IPF Explant	0.944	74%	100%	100%	65%	83%	< 0.0001

## Discussion

This study shows that IPF gene signatures can be derived from whole lung tissue, given appropriate biospecimen selection and acquisition. In fact, this study serves as a proof-of-principle: mathematical models such as BPR (that handle high-dimensional data) can be used to develop multi-gene biomarkers for non-neoplastic lung disease, starting from gene expression profiles.

We profiled gene expression from whole lung in 11 patients with IPF and 6 normal controls. Samples of IPF were obtained during diagnostic surgical lung biopsies or during lung transplantation procedures. Whenever possible, we obtained samples from two different lobes of the lung. During the initial data processing phase of our analysis, we made several interesting discoveries. We found that gene expression is similar between different lobes of the lung (upper and lower) sampled from the same patient. We also found that gene expression differs substantially between IPF samples obtained at the time of biopsy versus explant.

Then we developed three gene expression models, designed for the diagnosis of IPF. These models were designed for functionality and portability: they were designed to predict the diagnosis of IPF across different patient populations and across different microarray platforms. Therefore, we needed to test our models on an independent cohort of samples containing both IPF and normal lung, to see if the models' predictions were accurate. This represents the first reported attempt to show validity of IPF gene expression signatures as diagnostic models.

We found that all three of our IPF gene expression signatures exhibited discriminatory power and could be used to predict a diagnosis of IPF (see Wilcoxon rank sum, Table 2). However, the signature derived from explanted samples was the most accurate at diagnosing IPF in this particular validation cohort. We postulate several explanations. First, our "IPF Explant" training cohort is probably the most similar cohort as compared with the validation cohort, which is highly enriched with explant and autopsy samples. Second, the homogeneity of samples in the "IPF Explant" cohort promotes a more discriminative model, given the available sample size; while the clinically heterogeneous "All IPF" and "IPF Biopsy" cohorts tend to develop less discriminative models. Finally, predictive accuracy of our models is linked to the prevalence of IPF in the validation cohort. These factors must be considered in the design of more definitive studies.

The fact that a homogeneous "IPF Explant" cohort is most robust highlights the inherent heterogeneity in the general IPF population (represented by "IPF Biopsy") and supports the need for better diagnostic tools.

In the past, other investigators examined gene expression from the lungs of patients with pulmonary fibrosis. Studies were designed to detect gene expression that was altered in pulmonary fibrosis [24-26]. Experiments were also designed as a means to elucidate mechanisms of pathogenesis or identify novel targets for therapy [27]. One problem with these older studies is the lack of replication in independent cohorts [28]. More recent studies focus on differential gene expression between clinical phenotypes such as acute exacerbations of IPF versus stable IPF [15,29,30]; and IPF versus hypersensitivity pneumonitis (HP) [31]. Yet, no study to date has presented a functional gene-based diagnostic model.

We acknowledge the limitations of our study. Our provisional models range from 63-83% accurate. The present study was performed on a small cohort and was only intended as a proof-of-principle. However, we believe that, by increasing the number of samples in our training cohort, we can refine the diagnostic model and increase the accuracy of diagnostic predictions. We also recognize the need to discriminate IPF from other subtypes of pulmonary fibrosis. Therefore, a definitive investigation must compare IPF gene expression with gene expression profiles of NSIP, HP and other subtypes of pulmonary fibrosis. Since BPR models are restricted to binary classifications, we would potentially extend the Bayesian SVD approach to multinomial outcomes, or other commonly employed methods for high-dimensional expression data (e.g., Classification and Regression Trees [CART]).

## Conclusions

We show that BPR is a powerful tool for developing gene signatures from non-neoplastic lung tissue. We hope that this study will lead to the development of a definitive diagnostic gene signature for IPF. To do this, it will be necessary to collect a larger cohort of high-quality biospecimens. We suggest that BPR can also be used to develop a prognostic gene signature for IPF by training a model with samples of rapidly progressive IPF versus slowly progressive IPF. Furthermore, we believe that BPR can be used to model other lung disorders (such as NSIP, HP, bronchiolitis obliterans) by substituting with different phenotypes in the training cohort.

## Competing interests

The authors declare that they have no competing interests.

## Authors' contributions

EBM participated in the diagnosis and recruitment of the training cohort; clinical data analysis; microarray data analysis; statistics; conceptualization, planning and design of the study; and manuscript preparation, including preparation of the initial draft. WTB participated in the statistical design of the study, microarray data analysis and manuscript preparation. TAD, RDD, SSL, MWO and LDW participated in patient recruitment and development of the tissue acquisition protocol. TAS participated in histopathological review of the specimens; and participated in the development of the tissue procurement protocol. MPS participated in patient recruitment, development of the tissue procurement protocol and manuscript preparation. PWN participated in the diagnosis and recruitment of the training cohort; conceptualization, planning and design of the study design; and manuscript preparation. All authors read and approved the final manuscript.

## Pre-publication history

The pre-publication history for this paper can be accessed here:

http://www.biomedcentral.com/1755-8794/4/70/prepub

## Supplementary Material

Additional file 1 **Supplemental Methods**. Complete summary of the statistical methods and data integration steps used to develop and validate the multi-gene models. Click here for file

Additional file 2 **Model Selection (Figure S1)**. In order to optimize the fitted models for IPF Biopsies and IPF Explants, (A) and (C) the total sum of deviance was calculated for the observed phenotype versus posterior probabilities, and (B) and (D) the misclassification rate was computed under leave-one-out re-sampling for model sizes from 50 to 250 genes. Click here for file

Additional file 3 **Mapping the *ALL IPF *Gene Signature to GSE10667 (Table S1)**. 148 out of 151 (98.0%) possible features from the training dataset were mapped to corresponding features of the validation dataset on a many-by-many basis. Click here for file

Additional file 4 **Mapping the *IPF Biopsy *Gene Signature to GSE10667 (Table S2)**. 151 out of 153 (98.7%) possible features from the training dataset were mapped to corresponding features of the validation dataset on a many-by-many basis. Click here for file

Additional file 5 **Mapping the *IPF Explant *Gene Signature to GSE10667 (Table S3)**. 69 out of 70 (98.6%) possible features from the training dataset were mapped to corresponding features of the validation dataset on a many-by-many basis. Click here for file

Additional file 6 **Software codes in the R programming language (Bioconductor)**. Includes the algorithm for Bayesian Probit Regression. These codes are written for a specific machine. Please contact the authors for instructions on how to run these codes on another machine. Click here for file

Additional file 7 **Differentially Expressed Genes, IPF Biopsies versus IPF Explants (Table S4)**. Between IPF biopsies and IPF explants, 13 probesets, corresponding to11 unique genes, are differentially expressed at a FDR threshold of 10%. A positive t-statistic indicates up-regulation in the explants relative to the biopsies. Click here for file

Additional file 8 **Complete Gene List for the *All IPF *Model (Table S5)**. The top 151 probe sets identified by Student t-test correspond to 136 unique genes. A positive t-statistic indicates up-regulation in IPF relative to Normal. Click here for file

Additional file 9 **Complete Gene List for the *IPF Biopsy *Model (Table S6)**. The top 153 probe sets identified by Student t-test correspond to 131 unique genes. A positive t-statistic indicates up-regulation in Biopsies relative to Normal. Click here for file

Additional file 10 **Complete Gene List for the *IPF Explant *Model (Table S7)**. The top 70 probe sets identified by Student t-test correspond to 65 unique genes. A positive t-statistic indicates up-regulation in Explants relative to Normal. Click here for file
