# Supplementary material for: Bayesian probit regression model for the diagnosis of pulmonary fibrosis: proof-of-principle
Source: BMC Med Genomics. 2011 Oct 5;4:70. doi: 10.1186/1755-8794-4-70 (PMC3199230; doi:10.1186/1755-8794-4-70)
Supplement: Additional file 1 — Supplemental Methods. Complete summary of the statistical methods and data integration steps used to develop and validate the multi-gene models. [file 1755-8794-4-70-S1.DOC]

**Supplemental Methods**

The following sections outline the statistical methods, and data integration steps that were taken to generate all figures and results in “Bayesian Probit Regression Model for the Diagnosis of Pulmonary Fibrosis: Proof-of-Principle”, pertaining to the development and validation of multi-gene models for IPF. All microarray pre-processing, BPR modeling and analyses were performed using R version 2.9, and the additional packages from CRAN and Bioconductor: gplots, scatterplot3d, ROCR, safe, limma, and hgu133plus2.db. Functions for data normalization and Bayesian multi-gene modeling are available by request from Dr. Barry.

**Bayesian Probit Regression (BPR)**

Predictive models were derived from microarray data using an established method of data decomposition and Bayesian probit regression, as described in West et al (2001)[^1^](#_ENREF_1) and Mendiratta et al (2009).[^2^](#_ENREF_2) Briefly, using the training data alone, the features of the model were selected as the probe sets having the strongest (Pearson) correlation to phenotype tabulated above. Expression values were summarized by the top two principal components from a singular value decomposition derived from training samples alone. Summarized expression values (termed ‘metagenes’ in previous publications) are applied to a Bayesian probit regression model with non-informative priors for the parameters pertaining to the linear model and variance term. A Markov Chain Monte Carlo (MCMC) is used to obtain the posterior distribution for the linear predictor and regularized probabilities for each decomposed data set, and a numerical seed specified prior to each iterative algorithm such that results are fully reproducible from run to run. As an internal evaluation of performance ***during model development*** we conducted a leave-one-out cross-validation (LOOCV) of the model, whereby feature selection is repeated for each sample and the expected predicted probability is taken as the average value from the posterior distribution derived from the MCMC.

**Model optimization**

In order to tune parameters in the multi-gene models of IPF to give the largest separation between binary phenotypes, a data-driven empirical approach was taken to select the optimal number of features in each gene signature. For each model, the discriminatory power is evaluated using the misclassification rate under LOOCV (using an a priori defined threshold of Pr ≥ 0.5), and secondarily, the sum of deviances between observed and predicted phenotypes from the model (**Additional file 2, Figure S1**). For equivalent model performance, a larger gene-set is chosen in order to be more robust when validating in cross-platform analyses with incomplete mapping (see below). Note: because the LOOCV performance is used to optimize the multi-gene models, an independent validation is required to fully assess their predictive value.

**Mapping Affymetrix-derived signatures to the Agilent platform**

To independently validate the multi-gene models, features were mapped on a many-by-many basis between the training dataset (Affymetrix HGU133 Plus 2.0) and GSE10667 dataset (Agilent-014850 Whole Human Genome 4x44K Microarray) using the union of Unigene and RefSeq IDs provided with the external dataset in GEO and obtained from the Bioconductor package hgu133plus2.db version 2.2.11. In order to merge the datasets derived from Affymetrix and Agilent platforms when using the ‘many-to-many’ mapping created by the gene annotation, we selected the highest expressed feature on the Agilent platform as an unbiased indicator of stability in the gene expression estimates (Barry et al 2010).[^3^](#_ENREF_3) For the *All IPF* gene signature (Affymetrix HGU133 Plus 2.0), the result was that 148 out of 151 probesets from the *All IPF* gene signature were mapped (98.0%). For the *IPF Biopsy* gene signature, the result was that 151 out of 153 probesets from the *IPF Biopsy* gene signature (98.7%) were mapped. For the *IPF Explant* signature, the result was that 69 out of 70 probesets from the *IPF Explant* signature (98.6%) could were mapped (**Additional files 3-5,** **Tables S1-S3**).

**Normalization of training and test datasets**

Prior to independent validation, normalization steps are required to accommodate global differences in gene expression between the two array-platforms (Quackenbush, 2002).[^4^](#_ENREF_4) A critical modification to the normalization procedures was made in order for the features of the gene signatures to remain constant when applied to multiple validation sets. For each procedure, the gene expression data in the test set are transformed to the profiles of the training dataset (i.e. mean and variance of expression for each gene standardized) rather than a common profile. Without this modification to the common approaches of data normalization, the feature selection and expression data that informs each genomic signature may be influenced simply by the steps taken in merging with test datasets. Finally, because of the incomplete mapping across platforms, the SVD factors must be recomputed and applied to the BPR model process. However, the loadings to the factors are computed from training data only, such that test data are projected into this space. Further, no additional tuning of model parameters is done. Therefore, the test set has no influence on the Bayesian regression model, as compared to previous applications of the model (Potti, 2006 – now retracted)[^5^](#_ENREF_5) which have been criticized for, among other issues, having information leaking from the test set into the model development process potentially biasing the result (Baggerly 2007).[^6^](#_ENREF_6)

1. West, M.*, et al.* Predicting the clinical status of human breast cancer by using gene expression profiles. *Proceedings of the National Academy of Sciences of the United States of America* **98**, 11462-11467 (2001).

2. Mendiratta, P.*, et al.* Genomic strategy for targeting therapy in castration-resistant prostate cancer. *J Clin Oncol* **27**, 2022-2029 (2009).

3. Barry, W.T.*, et al.* Intratumor Heterogeneity and Precision of Microarray-Based Predictors of Breast Cancer Biology and Clinical Outcome. *J Clin Oncol* **28**, 2198-2206 (2010).

4. Quackenbush, J. Microarray data normalization and transformation. *Nat Genet* **32 Suppl**, 496-501 (2002).

5. Potti, A.*, et al.* Genomic signatures to guide the use of chemotherapeutics. *Nat Med* **12**, 1294-1300 (2006).

6. Coombes, K.R., Wang, J. & Baggerly, K.A. Microarrays: retracing steps. *Nat Med* **13**, 1276-1277; author reply 1277-1278 (2007).
