# Supplementary figures and images for: Bayesian probit regression model for the diagnosis of pulmonary fibrosis: proof-of-principle
Source: BMC Med Genomics. 2011 Oct 5;4:70. doi: 10.1186/1755-8794-4-70 (PMC3199230; doi:10.1186/1755-8794-4-70)

A

IPF Biopsy - fitted

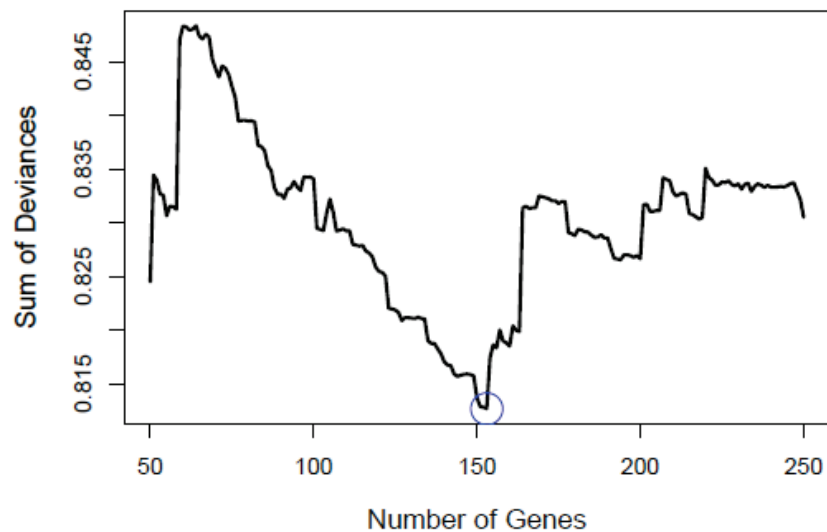

B

IPF Biopsy - Leave-one-out

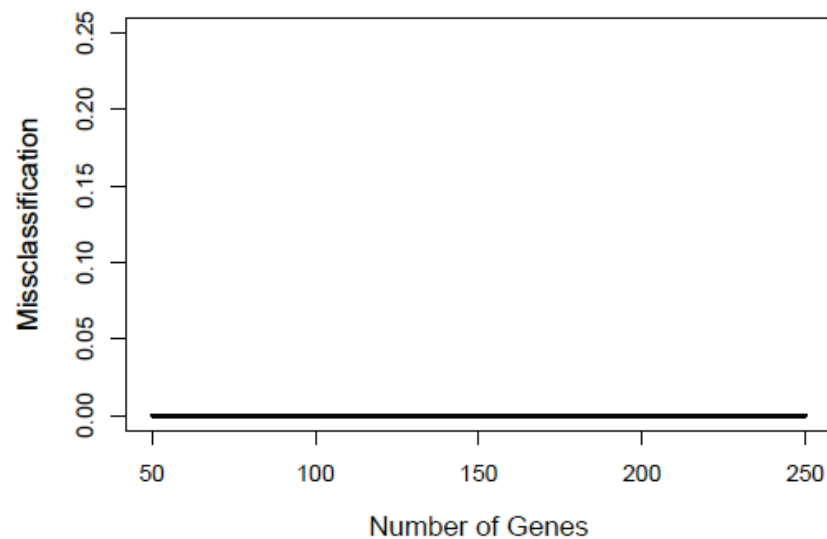

C

IPF Explant - fitted

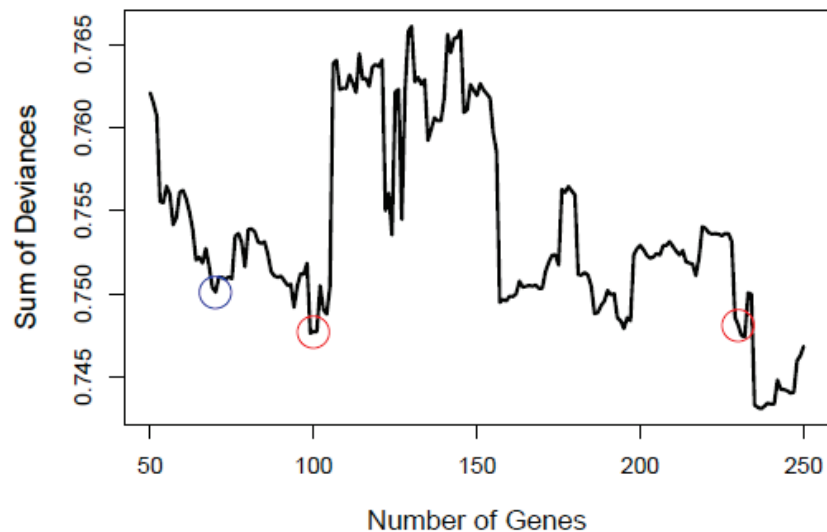

D

IPF Explant - Leave-one-out

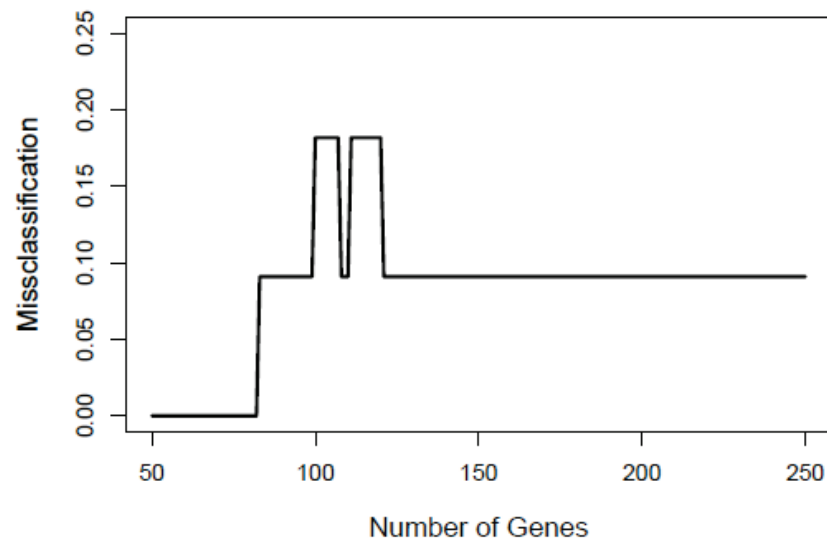

Supplement: Additional file 2 — Model Selection (Figure S1). In order to optimize the fitted models for IPF Biopsies and IPF Explants, (A) and (C) the total sum of deviance was calculated for the observed phenotype versus posterior probabilities, and (B) and (D) the misclassification rate was computed under leave-one-out re-sampling for model sizes from 50 to 250 genes. [file 1755-8794-4-70-S2.PDF]
